# Neuroplastic Effects in Patients With Traumatic Brain Injury After Music-Supported Therapy

**DOI:** 10.3389/fnhum.2019.00177

**Published:** 2019-06-25

**Authors:** Berit Marie Dykesteen Vik, Geir Olve Skeie, Karsten Specht

**Affiliations:** ^1^Department of Biological and Medical Psychology, University of Bergen, Bergen, Norway; ^2^Department of Neurology, Haukeland University Hospital Bergen, Bergen, Norway; ^3^Grieg Academy Research Centre of Music Therapy (GAMUT), University of Bergen, Bergen, Norway; ^4^Department of Education, UiT/The Arctic University of Norway, Tromsø, Norway; ^5^Mohn Medical Imaging and Visualization Centre, Haukeland University Hospital Bergen, Bergen, Norway

**Keywords:** social interaction, emotional control, neuroplasticity, mTBI, orbitofrontal cortex, music-supported therapy, dynamic causal modeling

## Abstract

Damage to the orbitofrontal cortex (OFC) often occurs following a traumatic brain injury (TBI) and can lead to complex behavioral changes, including difficulty with attention and concentration. We investigated the effects of musical training on patients with behavioral and cognitive deficits following a mild traumatic brain injury (mTBI) and found significant functional neuro-plastic changes in the OFC’s networks. The results from neuropsychological tests revealed an improved cognitive performance. Moreover, six out of seven participants in this group returned to work post intervention and reported improved well-being and social behavior. In this study, we explore the functional changes in OFC following music-supported intervention in reference to connecting networks that may be responsible for enhanced social interaction. Furthermore, we discuss the factor of dopamine release during playing as an element providing a possible impact on the results. The intervention consisted of playing piano, two sessions per week in 8 weeks, 30 min each time, with an instructor. Additional playing was required with a minimum of 15 min per day at home. Mean time playing piano in reference to participant’s report was 3 h per week during the intervention period. Three groups participated, one mTBI group (*n* = 7), two control groups consisting of healthy participants, one with music training (*n* = 11), and one baseline group without music training (*n* = 12). Participants in the clinical group had received standardized cognitive rehabilitation treatment during hospitalization without recovering from their impairments. The intervention took place 2 years post injury. All participants were assessed with neuropsychological tests and with both task and resting-state functional magnetic resonance imaging (fMRI) pre-post intervention. The results demonstrated a significant improvement of neuropsychological tests in the clinical group, consistent with fMRI results in which there were functional changes in the orbitofrontal networks (OFC). These changes were concordantly seen both in a simple task fMRI but also in resting-state fMRI, which was analyzed with dynamic causal modeling (DCM). We hypothesized that playing piano, as designed in the training protocol, may provide a positive increase in both well-being and social interaction. We suggest that the novelty of the intervention may have clinical relevance for patients with behavioral problems following a TBI.

## Introduction

Traumatic brain injury (TBI) can be devastating and cause changes in a human’s life. Optimal treatment and rehabilitation of patients with head injuries can mean the difference between disability or normal functioning. Despite the potential high relevance for the practice and management of mild traumatic brain injury (mTBI), few controlled studies have been carried out on the impact of long-term post-traumatic interventions (Heskestad, 2017). Well-designed studies of the outcome and efficacy of interventions in general, and in promoting patients with mTBI to return to work, is still lacking (Nygren-de Boussard et al., 2014). Vikane (2016) concluded in a longitudinal study of patients with mTBI, that multidisciplinary outpatient clinical treatment did not have a positive effect on whether the patient return to work (RTW) or stay on sick-leave. These results indicated that subsequent intervention studies should consider a different approach to promote RTW (Vikane, 2016).

Injury to the orbitofrontal cortex (OFC) alone, or combined with temporal pole damage, can result in complex behavioral changes (Mah et al., 2005). The orbitofrontal networks are in close proximity to bony protrusions and are vulnerable to trauma-induced rotational acceleration of the brain (Clark et al., 2018). Disturbances of higher cognition and social behavior have long been recognized as a common sequelae of lesions of the prefrontal cortex (Mah et al., 2005). Emotional recognition is important in social interactions, helping individuals to understand intentions and thereby guide behavior (Drapeau et al., 2017). The OFC receives input from the temporal association cortex, amygdala, and hypothalamus, making it the highest integration center for emotional processing. It also receives inputs from the visual system, taste, and somatosensory regions (Rudebeck et al., 2013). Behavioral changes have been linked with damage specifically involving OFC (Mah et al., 2005). Milder injuries to the head may cause microscopic damage to axons and thereby affect interconnected processing in the brain (Sigurdardottir, 2010). The injury may include white matter damage from a diffuse axonal injury (DAI), which can remain undetectable on structural neuroimaging (Balanger et al., 2007; Clark et al., 2018). A common impairment after mTBI is post-concussion syndrome. These often exist as a combination of symptoms and are categorized within three groups: cognitive problems (concentration and memorization); somatic symptoms (headache, vertigo, balance-problems, nausea, fatigue, sleep disorders and double vision problems) and behavioral problems such as an irritability, depression and anxiety, as well as response inhibition. These factors may have a negative impact on the individual’s social outcome (Heskestad, 2017).

The intervention method presented may be categorized within Neurologic Music Therapy (NMT), defined as “the therapeutic application of music to cognitive, sensory, and motor dysfunction due to neurologic disease of the human nervous system” (Thaut, 2010). The NMT method is based on neuroscience models of music perception and the influence of music on changes in non-music related brain areas. However, the present study does not strictly follow one of the cognitive techniques outlined in NMT. The intervention program is structured with a standardized curriculum.

### Why Is Music so Special and How Does Making Music Achieve Its Rehabilitation Effects?

A musical performance is a demanding task for the brain, involving the interaction of several modalities recruiting almost all regions of the brain (Koelsch and Siebel, 2005; Parsons et al., 2005; Zatorre, 2007; Jäncke, 2009). Learning to play an instrument requires complex multimodal skills involving simultaneous perception of several sensory modalities: auditory, visual, and somatosensory as well as those of the motor system (Pantev, 2009). Music-making provides an enriched environment for the brain by promoting dendritic sprouting that is fundamental for synaptic plasticity (Goldberg, 2009). Increasing evidence suggests that music making could be used as a possible tool in neurologic rehabilitation (Jäncke, 2009; Särkamö et al., 2014; Herholz et al., 2016). The psychological effects and neurobiological mechanisms underlying the effects of music interventions are likely to share common neural systems for reward, arousal, affect regulation, learning, and activity-driven plasticity (Sihvonen et al., 2017).

### What May be the Underlying Cognitive Processes Leading to Functional Neuroplasticity in OFC When Playing the Piano?

There is no specific music center in the brain. Different factors of music such as pitch, tempo, melody and dynamics are perceived in different neural networks (Parsons et al., 2005). Playing an instrument promotes an interaction between the two hemispheres and may strengthen the connections between neural networks (Schlaug, 2009b).

Based on knowledge from music psychology, music perception and cognition, as well as the effects of music production on changes in neural networks, we designed a music supported intervention to explore the behavioral and neuronal changes in patients with cognitive deficits following a mTBI (Vik et al., 2018). In this study, we were particularly interested in examining whether and how the intervention affects social interaction and behavior with a focus on the OFC as an area involved in social cognition but also most often damaged in TBI patients.

The previous report in Vik et al. (2018) demonstrated an interaction effect within OFC, where patients showed a specific increase of activity, compared to healthy controls. The present report explores this effect even further by examining explicitly which areas showed an increase in the patient, compared to both control groups. There is little knowledge about the potential neuroplastic changes induced by musical training within the emotional control and social enhancement of mTBI patients. According to a literature review by Hegde (2014), there are no studies that examine the improved functions over time. We may, therefore, suggest that this study fills a gap in the existing literature. We provide knowledge of how and why music-supported interventions for patients with deficits in social behavior may experience a positive enhancement of social interactions and thereby live a better life. The aim of the present study is to investigate factors during piano training that may be responsible for enhanced social interaction in patients with social deficits, following mTBI. We predict that piano training may enhance emotional control, thereby improving social behavior.

## Materials and Methods

### Participants

For this study, seven volunteers (three females) with cognitive deficits following mTBI were recruited with the help of the Department of Physical Medicine and Rehabilitation, Haukeland University Hospital, Bergen, Norway. Two control groups of healthy participants were recruited through posters at The University of Bergen and Haukeland University Hospital Bergen, Norway; one with music intervention (*n*_K1_ = 11), and one baseline group without music (*n*_K2_ = 12). All participants were aged between 18 and 60 years with a mean age of 38 years and a group matched for gender, age and education level. Inclusion criteria were 2 years post injury to avoid spontaneous recovery processes during the intervention period. Participants had received rehabilitation treatment prior to the intervention without any improvement of their deficits and had persistent problems with attention, concentration, memorization, fatigue, and social behavior, and were either on sick-leave, or working part time with adjusted work; see Table 1 for the clinical data of the patient group. We did not include a control clinical group. The reason for this was that these patients had received cognitive training and had participated in rehabilitation programs during hospitalization without any progress. We found that since the time post-injury was at least 2 years, we could avoid the confounding variable of spontaneous recovery, which takes place within the first month or year post injury. A longitudinal study of patients following mTBI supports this view. The authors concluded that for the group of patients with persistent symptoms at 2 months post-mTBI, this result represented a negative predictor for RTW (Vikane, 2016). Excluding parameters in all three groups were psychological problems before the accident. Musicians were excluded because of possible differences in brain structure between musicians and non-musicians (Stewart, 2008). Classification of musicians was that only persons with no formal training of music were accepted. All participants signed a consent form. The protocol was approved by Regional Ethical Committee (REK-Vest), Norway.

**Table 1:** Data of the clinical group.

Time to first examination after injury	Gender, Age	MRI/CT scan	Prior Intervention <2 years post injury	CVLT scores pre-post intervent.	Social behavior pre interv. and wellbeing	Work situation pre intervention	Work situation post intervention
Examination date: 2 months after concussion	P1, M42	Orbit and nose fracture	Outpatient Rehab.	17–60	Depressed. Isolated.	Sick leave 100%.	100% sick leave. Psychological problems
Examination date: 3 months after concussion	P2, F41	N	Outpatient rehab.	58–65	Depressed. Fatigue, isolated.	Sick leave 50%. Change of work adjusted to PTA.	Return to previous work 100%.
Examination date: 2 1/2 month after contusions	P3, M31	Small epidural hematoma and contusion bleeds frontal lobe right side and both temporal lobes	1 month in TBI unit	60–60	Sleep-disorders, fatigue.	Adjusted work following PTA	Return to previous work 100%.
Examination date: 20 days after concussion	P4, F52	Normal (Acach.cyst)	Outpatient TBI unit Rehab. 4 weeks	54–70	Avoiding gatherings, fatigue.	Adjusted work following PTA	Return to previous work 100%.
Examination date: 2 months after subdural hematoma (conservative management)	P5, F55	Orbit fracture and nose fracture	Outpatient TBI unit	32–48	Fatigue	Adjusted work following PTA	Return to work 100%.
First incidence 21 years prior to second incidence. Second incidence: Hospitalized. Examination date: 14 years after 2nd incident.	P6, M30	MR and CT micro bleeds contusions in frontal and temporal lobes bilateral 1st incidence. 2nd examination: MR.Post-traumatic changes in frontal- and temporal lobes.	TBI unit 1 month	62–74	Isolation after 1st incident. Difficult in decoding people’s faces and intensions. No changes post-intervention	Sick-leave 100%	Change of work. 100% work.
Examination date: 1 month after evacuated epidural hematoma	P7, M19	Diffuse axonal injury, Epidural hematoma contusions	TBI unit	60–70	Pre-:fatigue. Post- interv. Improved social interaction	Difficulties in following school-work due to fatigue.	100% back to school. Started study university.

*Clinical data was obtained from patient’s medical records, neuropsychological test results from a California Verbal Learning Test (CVLT) test and, social behavior and wellbeing prior to intervention was obtained from semi-structured interviews. Data on the work situation prior and post-intervention was also obtained from semi-structured interviews. All patients have been diagnosed with mild traumatic brain injury (mTBI) according to scores obtained in the Rivermead post-concussion symptoms questionnaire and The Glasgow Coma Scale. Scores are not included in this figure*.

### Study Design

We designed a between-group and a longitudinal within-subject design. All groups were examined pre-post intervention. Participants in the baseline group were examined without intervention in between. We applied neuropsychological tests as described in the following; neuroimaging, semi-structured interviews in the patient group only, self-report, training log and neuroimaging for all groups.

### Piano Training Protocol

The music groups received two 30 min, one on one piano-lessons per week, for 8 weeks. They were instructed to practice at home for a minimum of 15 min a day. The tuition repertoire consisted of 28 pieces for beginners; see Vik et al. (2018) for a detailed tuition program. The curriculum consisted of learning to play melodies, first with each hand separately, then after learning to play two octaves, playing with both hands simultaneously. The curriculum can be found in the Norwegian piano-tuition book for beginners in which the first 28 pieces served as the curriculum (Agnestig, 1958). Reading musical notation, theory and playing scales were included in the curriculum. Additional pieces were provided if the participant had reached the 28 pieces before the end of the 8 weeks of training. We did not evaluate the level of performance, because the main focus was on actual training time. An important aspect of the lesson was a repetition of previous lesson’s objectives to facilitate neuroplasticity as described in the “Introduction” section. We, therefore, performed a log of the participant’s attendance. The instructor of the intervention, was an experienced piano teacher with a Masters in Piano Pedagogy and in Music Psychology, with over 25 years of teaching experience. The piano-training took place at Haukeland University Hospital, Bergen, Norway.

Participants in both music groups kept a log of their actual training-time. The clinical group was instructed to report possible headache, vertigo or other somatic issues while playing the piano. Training-time could be an important variable in analyzing the results in reference to possible functional and structural changes in the brain’s neural networks, between the music groups and within the clinical group.

### Assessment Pre-post

#### Semi-structured Interviews

Semi-structured interviews were conducted pre-post intervention in the patient group. A summary of the answers can be found in Table 1. Of special interest was their subjective opinion of well-being, social interaction and cognitive performance pre-post intervention.

#### Neuropsychological Tests

A test-battery of three different neuropsychological tests was applied: Mini Mental Status Test (MMS), California Verbal Learning Test (CVLT 2), Stroop Word/Color test. Data were analyzed with a 3 (group) × 2 (repetition) repeated-measure analysis of variance (ANOVA), using SPSS 22^1^.

#### Functional and Structural MRI

Functional and structural magnetic resonance imaging (MRI) scans were collected-pre-post for all groups; for detailed information see Vik et al. (2018). In short, participants were examined on a 3T GE Signa MR scanner. They performed a passive listening task in which they listened to extracts of classical and popular Western musical cadence pattern of Tonika-Dominant-Tonika (TDT) chords, based on the first, the fifth, and the first tone of the scale. The relationship between individual tones forms the basis for the most fundamental aspect of musical processing and recognition of melodies (Zatorre, 2007). TDT is a melodic pattern in Western music activating semantic networks that are important for long-term representations of familiar tunes (Zatorre, 2007). This paradigm was selected because all participants were Norwegian, and Western music has a familiar structure that will activate association cortices. A key factor in the listening task is to activate most parts of the brain. As all melodic perception involves a working memory mechanism, a stronger involvement of the prefrontal cortex and other association cortices was predicted. The paradigm was adapted from Zatorre (2007) and is defined as a high cognitive task.

In total, 28 extracts from melodies were presented with varying delays between each stimulus as an event-related design. In total, 160 echo-planar imaging (EPI) images were acquired with the following parameters: repetition time (TR) 3 s; echo time (TE) 30 ms; matrix size 128 × 128; 30 slices; voxel size 1.72 × 1.72 × 4.4 mm. Data were analyzed using SPM12, and data processing included realignment, unwarping, normalization to the MNI reference space and smoothing (8 mm).

As described earlier, the initial 3 (groups) × 2 (repetitions) repeated-measure ANOVA, revealed a significant (FWE-corrected) group × repetition interaction for details (see Vik et al., 2018), showing an increased activity in OFC in patients, but not in the controls. In the present report, we aimed at exploring this effect even further by re-analyzing the data. A non-parametric approach was selected since the patient group was too small for ordinary linear statistics. Using the SnPM13 toolbox (Nichols and Holmes, 2001; Winkler et al., 2014), the two control groups were pooled and treated as one control group and compared to the patient group. This increases the specificity of those effects that occurred in the patient group only. A variance smoothing of 8 mm was applied and 5,000 permutations were estimated. The SnPM analysis was performed as a two-sample test with the pre-post difference images as an input, with the hypothesis that both groups are equal. The results were explored with a nonparametric *p*-value of *p* < 0.001 and only clusters with at least 10 voxels were considered. The pre-post difference images were estimated manually within SPM, using the ImCalc function. These data were also analyzed in conjunction with performance on cognitive tasks and are published separately (Vik et al., 2018). This analysis served as a precursor for the following dynamic causal modeling (DCM) analysis of the resting-state functional magnetic resonance imaging (rs-fMRI) data. Therefore, and since the sample size in this study is low (seven patients with mTBI and complete dataset), we selected an explorative analysis with a liberal statistical threshold for the fMRI results (10 voxels). This resulted in seven areas of interest, which were then entered into the rs-fMRI analysis.

#### Resting-State fMRI

Resting-state fMRI data were also acquired both in the pre- and post-session. The rs-fMRI examination lasted almost 9 min, and the following parameters were used: 256 EPI images; TR 2 s; TE 30 ms; matrix size 64 × 64; 30 slices; voxel size 3.44 × 3.44 × 4.5 mm. Data were pre-processed in the same way as described above. Prior to the analysis, the time series from the seven areas of interest were corrected for some global signal fluctuations. Therefore, fluctuations co-occurring in white matter and the ventricles were regressed out. The cleaned time series’ were analyzed with spectral dynamic causal modeling (spDCM) for resting state, with the seven areas of interest (Friston et al., 2014; Razi et al., 2015; Kandilarova et al., 2018). The spDCM model is a fully connected model, where each node is connected to each other node.

In contrast to a stochastic DCM on resting-state fMRI data, a spectral DCM estimates effective connectivity from the cross spectra of the fluctuations in neuronal states instead of from their time courses directly. Further, the individual spDCM models were not separately but jointly estimated, using the Parametric Empirical Bayes (PEB) framework, implemented in SPM12.2. This was followed by Bayesian model reduction to restrict the number of parameters and a second level PEB analysis, where different design-matrixes were tested against each other, and the model with the highest Bayesian model evidence was analyzed further. The different designs parameterized that there were no-effects, a pre-post effect (independent of groups), a pre-post effect only for training (for patients and the control group with training), general differences between the patient and control groups (across pre-post measurements), or group-specific effects. The overall network connectivity of the winning model was explored across groups and repetitions in terms of posterior probabilities (*P*p > 0.95) of the estimated parameter. For the analysis of interaction effects, the estimated parameter (A-matrix) were extracted from the individual spDCM models and further analyzed, using repeated-measure ANOVAs. Due to the small number of patients, the resulting interaction effects were further investigated with non-parametric Wilcoxon rank sum tests. To keep the number of performed test low, only group differences in pre-post changes in connectivity were analyzed.

## Results

### Emotional and Social Interaction—Semi-structured Interviews—Group 1 (Patient Group)

Data from semi-structured interviews demonstrate a qualitative increase in social interaction and well-being post-intervention (see Table 1). Common problems before intervention were fatigue, blurred vision, light sensitivity, dizziness, vertigo, headache and sensitivity to sound. Increased irritability, mood changes and withdrawal from social gatherings were also reported. This subjective information corresponds with the patient’s medical records from hospitalization. Post-intervention there were no reports of negative issues in reference to the intervention. On the contrary, all patients had a positive intervention experience. The answers corresponded with the individual training logs in which the participants were instructed to write notes of any changes in cognitive ability and social functionality during the intervention. Six out of seven of the participants reported progressive improvement of cognitive performance during the remaining 4 weeks of intervention and improved mental capacity after 8 weeks. However, two of seven patients did not improve their social behavior as reported in Table 1, P1 had developed post-traumatic depression and felt isolated, and P6 reported difficulties in emotional recognition in social settings, followed by poor social activity and a feeling of isolation, despite a positive work-situation post intervention.

### Training Time vs. Neuropsychological Test Results—Clinical Group

Training time may be a variable of interest. The question is if there was a relationship between training time, scores on the CVLT test and the final outcome. As we found functional changes in the OFC’s neural networks only in the clinical group, we have limited these analyses to this particular group. In Table 2 we can see there is no consistency between scores in CVLT, amount of training time and outcome in reference to RTW, Table 1. There were extensive individual differences of training time within this group. Mean training time was 3 h per week. P7, Table 2, played the minimum time per week for 8 weeks, namely 15 h and 20 min. This participant was present at all lessons and reported to follow the scheduled 15 min home-playing. He had a significant increase in CVLT scores and returned to his studies post-intervention, which he had not been able to attend after injury.

**Table 2:** Clinical group data.

Patient	CVLT 2 Pre-interv	CVLT2 Post-interv	Total training-time	
P 1 42 M	17	60	7 h 45 min
P 2 41 F	58	65	36 h 45 min
P 3 31 M	60	60	8 h
P 4 52 F	54	70	28 h 35 min
P 5 55 F	32	48	23 h 55 min
P 6 30 M	62	74	21 h 20 min
P 7 19 M	60	75	15 h 20 min

*Total training time and test-results from neuropsychological test CVLT pre- and post-intervention*.

P1 and P3 had a total training time below the set minimum time which was estimated as 15 h 30 min, for 8 weeks. P1’s total training time was 7 h 45 min which indicates no training at home between lessons with the instructor. P1 was present at all lessons except one. He self-reported to have developed a post-depression condition. He achieved a significant increase of scores in the CVL test post intervention. P3 did not provide any explanation for not following the intervention program strictly. This participant did not have any increase in scores from the CVL test.

### Neuropsychological Tests

As reported earlier, the CVLT test demonstrated a significant effect of musical training on executive functions related to attention, learning strategies, and retrieval of memories in the patient group and the healthy control group with music intervention. More precisely, in the post-intervention examination, the patient group demonstrated an increase in performance up to the level of the pre-intervention level of both control groups (for statistical details see Vik et al., 2018).

However, the Stroop test did not show any group-specific effects, but a main effect of repetition (for statistical details see Vik et al., 2018). The MMS test was excluded from this study because of a ceiling effect of all patients.

### fMRI-Tonika-Dominant-Tonika Task

**Figure 1:**
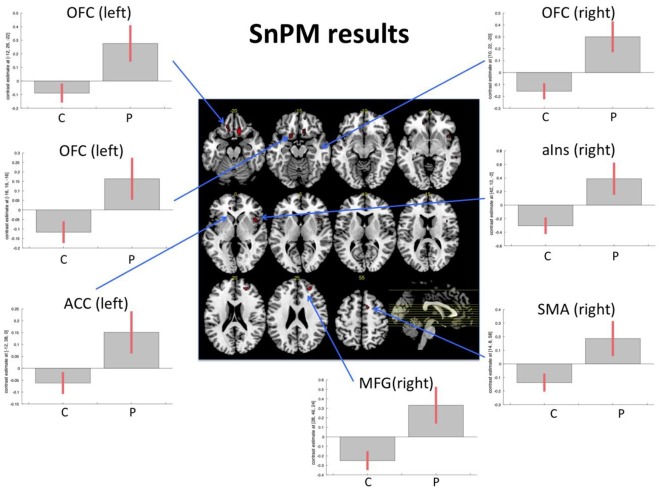
Functional magnetic resonance imaging (fMRI) task. Figure displays the results from the non-parametric analysis on the pre-post differences. The results highlighted areas where patients (P) demonstrated an increase of activity from pre- to post-examination, while control subjects (C; irrespective of music intervention) did not show an increase or even a slight decrease. The results are explored with a non-parametric *p*-value of *p* < 0.001. Bar plots show contrast estimates (with 90% confidence interval). OFC, orbitofrontal cortex; MFG, middle frontal gyrus; aIns, anterior insula; ACC, anterior cingulate cortex; SMA, supplementary motor area.

The nonparametric SnPM analysis was examined with a nonparametric *p*-value of *p* < 0.001 and only clusters with at least 10 voxels were considered. This revealed seven areas that showed an increase in the patient group only. These were the right medial orbitofrontal gyrus, middle frontal gyrus (MFG), anterior insular cortex, two distinct clusters within the left medial orbitofrontal gyrus (hereafter called left anterior OFC and left posterior OFC), the supplementary motor area (SMA), and the rostral anterior cingulate cortex (ACC). There were no areas showing a decrease in patients/increase in controls (Table 3, Figure 1).

**Table 3:** Table reports results from the nonparametric functional magnetic resonance imaging (fMRI) analysis with anatomical description, MNI coordinates, statistical values, and cluster size (number of voxels, voxel size 2 × 2 × 2 mm).

Area	Side	Co-ordinates	Pseudo T	*p* (non-para)	Cluster size
Medial orbitofrontal gyrus	R	10, 22, −20	4.88	0.0002	92
Middle frontal gyrus	R	28, 46, 24	3.96	0.0004	44
Anterior insular cortex	R	42, 12, −2	3.85	0.0004	52
Medial orbitofrontal gyrus (anterior)	L	−12, 26, −22	3.73	0.0002	25
Medial orbitofrontal gyrus (posterior)	L	−16, 16, −16	3.39	0.0004	25
Supplementary motor area	R	14, 8, 58,	3.24	0.0006	21
Rostral anterior cingulate cortex	L	−12, 38, 0	2.90	0.0002	14

*The results were explored at a nonparametric p threshold of p < 0.001 and only clusters with at least 10 voxels per cluster were considered*.

### rs-fMRI—Dynamic Causal Modeling

**Figure 2:**
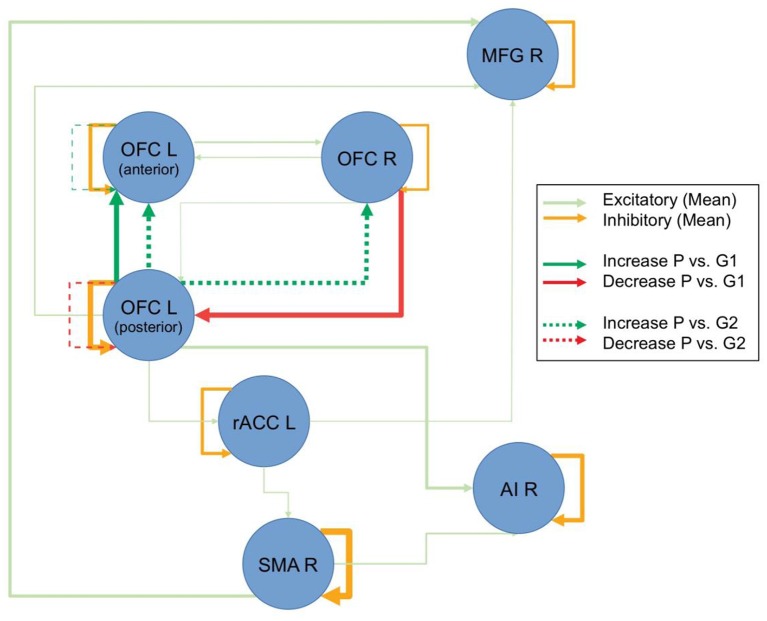
Resting-state dynamic causal modeling (DCM) analysis. Figure displays the overall connectivity of the discovered network in the shaded colors, independent of groups and repetitions. The line thickness for the shaded lines is an indicator of the averaged estimated parameter. Pre-post group differences are displayed in strong colors. Connections, where there was a difference between the patients (P) and the control group with intervention (G1) are displayed with continuous lines, and comparisons to the control group without intervention (G2) are displayed with broken lines. Line thickness inversely scales with the *p*-values (Note: line thicknesses of the lines in shaded and strong colors are not related and should only illustrate the relative strength of the respective effects).

The model with the highest Bayesian model evidence (relative log-evidence of BF = 11) was the one that parameterized a general group difference between the patients and control groups. To examine the overall underlying connectivity pattern, only connections with a posterior probability of *P*p > 0.95 were explored, as estimated by PEB, implemented in SPM12.2. This overall connectivity pattern of the investigated network is displayed with shaded colors in Figure 2. The repeated-measures ANOVA on the connection strength, revealed five connections where there was an interaction effect between the patient and control groups. The non-parametric Wilcoxon rank sum test revealed that the patient group primarily demonstrated a dominating change in functional connectivity. Compared to the control group that received the same training, patients showed reduced functional connectivity from the right OFC to the left posterior OFC and increased functional connectivity from the left posterior to the left anterior OFC (bold colored, continuous lines in Figure 2) after the intervention. When compared to the control group without training, the same increased functional connectivity between the two left-sided OFC nodes was found again, as well as an increased functional connectivity from left posterior to the right OFC, an increased self-inhibition of the left anterior OFC (i.e., less activity), and a reduced self-inhibition of the left posterior OFC (i.e., higher activity; broken lines in Figure 2). When comparing the two control groups, only an increased self-inhibition of the left anterior OFC and increased connectivity between the anterior insula and ACC was discovered for the control group with training (not displayed in Figure 2). Interestingly, without any restrictions of the analysis, all differential effects for the patient group were exclusively found for the OFC, while all other examined connections remained unchanged, independent of group, repetition, or perceived training.

## Discussion

The present results document functional changes in several dimensions. The qualitative outcome from semi-structured interviews in which six out of seven participants in the clinical group reported improved well-being and social interaction together with a normal work-situation, supports the view of increased social behavior after music intervention. This was accompanied with functional neuroplasticity predominantly in the orbito- and prefrontal cortex following music-supported intervention in the patient group, as concordantly seen in both task and resting-state fMRI. There may be several possible reasons regarding the clinical group’s improved social functioning, further discussed below.

The fact that playing a musical instrument is a complex activity for the brain and activates almost all region of the brain (Zatorre, 2007), may be an essential key factor in the enhancement of cognitive functions and thereby the improvement of well-being and social interaction. Evidence-based research on how music activates the brain supports this view. Schlaug (2009b) documented that the corpus callosum, the fiber-bundle between the two hemispheres, was larger in musicians than in non-musicians, demonstrating that musical training relies on inter-hemispheric brain networks.

Training time could be a variable. However, as seen in Table 2, there is no causal relationship between the amount of training time and the scores from CVLT. As reported earlier, P1 and P3 had a total training time below the set minimum time, which was estimated at 15 h 30 min over 8 weeks. P1’s total training time was 7 h 45 min which indicates no training at home between lessons with the instructor, as he was present at all lessons except for one. It is an open question why there is a gap between CVL test scores and an improvement of social interaction. One could speculate if attending lessons would enhance attention, concentration and memory function, but not have an impact on neuroplasticity in OFC and thereby enhanced social behavior. P3 differed from P1. P3 was not present more than every other lesson, however, he practiced at home and achieved 8 h of total practicing time according to a self-report. His scores on the CVL test did not differ from pre-post intervention. This could perhaps be interpreted as an importance of regular attendance during 8 weeks of intervention. Limitations to draw any conclusion of the impact of training time vs. neuropsychological tests scores are linked to the low participant numbers of the sample size.

It is interesting to register that the minimum training time of a total 15 h over 8 weeks indicates a norm for improved cognitive performance and social behavior. Future studies should replicate the training protocol of training time to establish a controlled method.

### How May Piano-Playing Improve Cognitive Functioning?

There are specific interconnected cognitive processes which may lead to functional neuroplasticity in OFC and behavioral changes during playing the piano. A number of studies have evidenced that there are shared neural networks between language and music (Patel et al., 1998; Koelsch and Siebel, 2005; Parsons et al., 2005; Brown et al., 2006). Music activity also activates areas involved in episodic and semantic memory networks (Chan et al., 1998; Schlaug, 2009a), and stimulates and strengthens perception and cognition by activating neural networks that are involved in analyzing perceptual patterns in music (Deutsch, 1982). Finally, repetition, evident in musical structures, may contribute to strengthening new neural connections through neuroplastic changes—a core mechanism of learning (Hebb, 1961; Ockelford, 1999; Münthe et al., 2002; Goldberg, 2009).

There are other aspects to consider in reference to neural activity during music production in reference to what may influence well-being and an enhanced social life. An intriguing question is if dopamine release during active and successful playing of a musical instrument could be responsible for the improvement of cognitive performance. This activity is goal oriented and mastering of a goal is a dopamine release factor (Lehrer, 2011). Music listening and production activate the reward circuitry cortical networks in reference to emotional reward, followed by a dopamine release—a neurotransmitter evident in the reward system (Owessen-White et al., 2016; Brodal et al., 2017). Dopamine may be a core factor for increased neuroplasticity during musical training. When playing an instrument, like a piano, there are certain factors working together in concert to achieve dopamine release: motivation to reach a goal and the satisfaction of mastering a goal. One could speculate that positive emotional responses to music cause an additional stimulation of the limbic system, followed by a dopamine release. The amygdala and association cortices may also be activated when processing those emotional responses (Tramo, 2001).

Last, but not least, the enjoyment of playing the piano, as reported by the participants, may have a possible dopamine-releasing effect, thereby increasing the neurotransmitter-effect between the neural networks affecting OFC and executive functions, followed by the normalization of emotional reactions that are fundamental in social interactions. Playing the piano has a profound effect on the neural networks in engaging neural circuits evident in emotion and reward, which is important for stimulus-reinforcement learning, an essential factor in social interaction (Rolls, 2014).

Further evidence comes from the two present fMRI analyses. When explicitly exploring the recovery in patients, the results demonstrated that besides the earlier reported functional change in the right OFC (see Vik et al., 2018) changes in the left-sided homolog, the right middle prefrontal cortex, right anterior insular cortex, left rostral ACC, and the right SMA (see Figure 1) also occurred. Importantly, the discovered effects within OFC have been confirmed and replicated by the resting-state fMRI data, using DCM (see Figure 2). The three explored areas of the left and right OFC mostly showed increased connectivity and increased activity, which corresponds to the findings from the task-related fMRI. The areas that showed signs of recovery are all areas related to attention and cognitive control, but also to social cognition (Zald and Raucht, 2010; Clark et al., 2018) Interestingly, nearly all detected brain areas that showed a recovery effect also received direct or indirect dopaminergic connections, like the prefrontal cortex and the anterior insular cortex (Christopher et al., 2013). This might provide further support to the earlier proposed assumption that actively playing a musical instrument might have a dopamine-releasing effect. More importantly, the changes in OFC connectivity, as discovered by the spDCM analysis, indicates that the connectivity within this network mostly increased and that the discovered recovery process was indeed restricted to this area of the explored network. Although the explored sample is rather small, this study concurrently demonstrated in two independent analyses and fMRI datasets, that a recovery process took place within the OFC. It is important to emphasize that both analyses were not restricted to the OFC and that both results emerged out of two unrestricted analyses. Future studies, however, have to replicate this finding with a larger sample of patients, before final conclusions can be drawn.

Finally, the increased activity of the bilateral OFC could be seen in light of the increased scores in social interaction. Interestingly, this present task fMRI analysis also revealed increased activation in the rostral anterior cingulate gyrus, which is also called the emotional part of the anterior cingulate gyrus and is closely related to error monitoring (Bush et al., 2000). Furthermore, the other areas from the task fMRI analysis that showed increased activations are mostly related to different attentional systems, with the anterior insula as the central area for the saliency network (Menon and Uddin, 2010), and the MFG as part of the central executive network (Corbetta et al., 2008; Hugdahl et al., 2015). The involvement of these areas in the rehabilitation processes, triggered by the intervention, may reflect that active music training goes beyond the simple training of new skills, but involves several brain areas and networks that have to interact, in addition to the fact that piano training is an activity with substantial social interaction. Although this was a mostly explorative analysis, we should mention that virtually all areas showed an increase of the levels of activations from pre- to post-intervention measurement in patients, while the control subjects showed almost no changes or a slight decrease (see Figure 1). However, given the size of the sample, this description remains of a more qualitative nature, and further studies with larger samples, with a control patient group to rule out which effects are dedicated to the music intervention and which effects might result from increased social interaction during and because of the training, are required.

## Conclusion

We have demonstrated that playing the piano may induce neuroplasticity and thereby enhance social interaction and well-being in patients with cognitive deficits following mTBI. The results from both task and resting-state fMRI revealed significant evidence for a causal relationship between music intervention and functional reorganization of neural networks in the OFC. The fact that six out of seven patients with chronic mTBI returned to work post-intervention is a promising outcome of this intervention. We suggest that neural activation during 8 weeks of intense and structured music intervention, promoted social interaction and enhanced cognitive performance in the clinical group, a view supported by the literature of neuroplastic changes in the brain during music-training. However, an interesting aspect of future research is to investigate the level of dopamine released during playing the piano in exploring the impact of dopamine on social behavioral, in reference to changes in OFC neural networks. A central limitation to the study is the low number of participants. Another limitation is the lack of a patient control group. Although the participants within the patient group had already received rehabilitation within the health system and were in a chronic phase of post-concussion syndrome, a control patient group would add more significance to the final results. Future studies should, therefore, include a patient control group. In conclusion, we propose that the novelty of this intervention may have clinical relevance for patients with problems in social interaction, following mTBI.

## Ethics Statement

The protocol was approved by Regional Ethical Committee (REK-Vest), Norway.

## Author Contributions

BV: doctoral thesis. GS: medical records. KS: MATlab fMRI.

## Conflict of Interest Statement

The authors declare that the research was conducted in the absence of any commercial or financial relationships that could be construed as a potential conflict of interest. The reviewer CG declared a shared affiliation, though no other collaboration, with the authors to the handling Editor.

## Footnotes

^1^www.ibm.com
